# Life Insurances

**Published:** 1882-05

**Authors:** J. B. Hodgkin


*Life Insurance.*—Several years ago the writer had occasion to read a paper before the Virginia State Dental Association on the above subject, the object being to stimulate the formation of a mutual association for the insurance of the lives of dentists. Nothing come of it however, as it seemed difficult to obtain any concert of action among the profession. Still, we think that the difficulties are not insuperable, and there is no reason why the dental profession of Virginia or any other State should not have an organization like that adopted by some of the clerical bodies of that State—a system by which a bereaved family is guaranteed a comfortable sum in case of the death of its head. Lately there has been organized in Staunton, Virginia, the Valley Mutual Life Association of Virginia, which has adopted some features somewhat new in this country in life insurance, though well known and long practised in England, features which cannot fail to be popular, because they are so eminently just. And we advise those of our professional bretheren who contemplate insuring their lives to examine the methods of this company. This is written without solicitation, or knowledge on the part of those managing this company, and simply because we feel that it presents advatages not given elsewhere. As a rule dentists, though handling money freely seldom lay up much, and it is easy for them tc pay out, from time to time the small sum necessary to keep up a policy which, in the event of death would keep wife and children from poverty.

J. B. Hodgkin.

